# Resilience after severe critical illness: a prospective, multicentre, observational study (RESIREA)

**DOI:** 10.1186/s13054-024-04989-x

**Published:** 2024-07-12

**Authors:** Alice Mathieu, Jean Reignier, Amélie Le Gouge, Gaetan Plantefeve, Jean-Paul Mira, Laurent Argaud, Pierre Asfar, Julio Badie, Nicolae-Vlad Botoc, Hoang-Nam Bui, Delphine Chatellier, Louis Chauvelot, Christophe Cracco, Michael Darmon, Agathe Delbove, Jérôme Devaquet, Louis-Marie Dumont, Olivier Gontier, Samuel Groyer, Yannick Hourmant, Samir Jaber, Fabien Lambiotte, Benjamin Madeux, Julien Maizel, Olivier Martinet, Virginie Maxime, Emmanuelle Mercier, Mai-Anh Nay, Saad Nseir, Gael Piton, Jean-Pierre Quenot, Anne Renault, Jean-Philippe Rigaud, Francis Schneider, Michel Sirodot, Bertrand Souweine, Fabienne Tamion, Didier Thévenin, Nathalie Thieulot-Rolin, Francois Tinturier, Patrice Tirot, Isabelle Vinatier, Christophe Vinsonneau, Jean-Baptiste Lascarrou, Alexandra Laurent, the NUTRIREA-3 Trial Investigators, the Clinical Research In Intensive Care and Sepsis (CRICS-TRIGGERSEP) Group

**Affiliations:** 1 Laboratoire de Psychologie: Dynamiques Relationnelles Et Processus Identitaires (Psy-DREPI), Université de Bourgogne Franche-Comté, EA7458, Dijon, France; 2 CHU Nantes, Movement - Interactions - Performance, MIP, UR 4334, Nantes Université, 44000 Nantes, France; 3 Médecine Intensive Réanimation, Nantes Université, CHU Nantes, 44000 Nantes, France; 4 Inserm CIC 1415, Tours, France; 5 Centre Hospitalier Universitaire de Tours, Tours, France; 6 Service de Médecine Intensive Réanimation, Centre Hospitalier d’Argenteuil, Argenteuil, France; 7 Service de Médecine Intensive Réanimation, Hôpital Cochin, Groupe Hospitalier Paris Centre-Université Paris Cité, Assistance Publique –Hôpitaux de Paris, Paris, France; 8 Service de Médecine Intensive Réanimation, Hôpital Edouard Herriot, Hospices Civils de Lyon, Lyon, France; 9 Service de Médecine Intensive Réanimation, Centre Hospitalier Universitaire Angers, Angers, France; 10 Service de Médecine Intensive Réanimation, Hôpital Nord Franche Comté, Trevenans, France; 11 Service de Médecine Intensive Réanimation, Centre Hospitalier de Saint Malo, Saint-Malo, France; 12 Service de Médecine Intensive Réanimation, Centre Hospitalier Universitaire de Bordeaux, Bordeaux, France; 13 Service de Médecine Intensive Réanimation, Centre Hospitalier Universitaire de Poitiers, Poitiers, France; 14 Service de Médecine Intensive Réanimation, Hôpital de la Croix Rousse, Hospices Civils de Lyon, Lyon, France; 15 Service de Médecine Intensive Réanimation, Centre Hospitalier d’Angoulême, Angoulême, France; 16 Université Paris Cité ; Assistance Publique –Hôpitaux de Paris, Service de Médecine Intensive Réanimation, Centre Hospitalier Universitaire Saint Louis, Paris, France; 17 Service de Réanimation Polyvalente, Centre Hospitalier Bretagne-Atlantique, Vannes, France; 18 Service de Réanimation Polyvalente, Hôpital Foch, Suresnes, France; 19 Service de Médecine Intensive Réanimation, Hôpital Louis-Mourier, Assistance Publique –Hôpitaux de Paris, Colombes, France; 20 Service de Médecine Intensive Réanimation, Centre Hospitalier de Chartres, Chartres, France; 21 Service de Médecine Intensive Réanimation, Centre Hospitalier de Montauban, Montauban, France; 22 CHU Nantes, INSERM, Nantes Université, Anesthesie Reanimation, CIC 1413, Nantes, France; 23 Service de Réanimation Chirurgicale, Hôpital Saint-Eloi, CHU de Montpellier, Montpellier, France; 24 PhyMedExp, INSERM, CNRS, Montpellier, France; 25 Service de Médecine Intensive Réanimation, Centre Hospitalier de Valenciennes, Valenciennes, France; 26 Service de Médecine Intensive Réanimation, Centre Hospitalier de Bigorre, Tarbes, France; 27 Service de Médecine Intensive Réanimation, Centre Hospitalier Universitaire Amiens-Picardie, Amiens, France; 28 Service de Médecine Intensive Réanimation, Centre Hospitalier Universitaire de la Réunion, Saint-Denis, La Réunion France; 29 Service de Médecine Intensive Réanimation, Hôpital Raymond Poincaré, Assistance Publique Hôpitaux de Paris, Garches, France; 30 Inserm U 1173, Université de Versailles-Saint Quentin en Yvelines, Versailles, France; 31 Service de Médecine Intensive Réanimation, Centre Hospitalier Universitaire de Tours, CRICS-TRIGGERSEP Network Tours, Tours, France; 32 Service de Médecine Intensive Réanimation, Centre Hospitalier Universitaire d’Orléans, Orléans, France; 33 Médecine Intensive-Réanimation, CHU Lille, 59000 Lille, France; 34 CNRS, Inserm, UMR 8576 - U1285 - UGSF - Unité de Glycobiologie Structurale et Fonctionnelle, Lille University, Lille, France; 35 Service de Médecine Intensive Réanimation, Centre Hospitalier Universitaire de Besançon, Besançon, France; 36 Université de Franche Comté, EA3920, Besançon, France; 37 Service de Médecine Intensive Réanimation, Centre Hospitalier Universitaire François Mitterrand, Dijon, France; 38 Lipness Team, INSERM, LabExLipSTIC, Université de Bourgogne, Dijon, France; 39 INSERM Centres d’Investigation Clinique, Département d’épidémiologie Clinique, Université de Bourgogne, Dijon, France; 40 Service de Médecine Intensive Réanimation, Centre Hospitalier Universitaire La Cavale Blanche, Brest, France; 41 Service de Médecine Intensive Réanimation, Centre Hospitalier de Dieppe, Dieppe, France; 42 Service de Médecine Intensive Réanimation, Hôpitaux Universitaires de Strasbourg, Strasbourg, France; 43 Service de Médecine Intensive Réanimation, Centre Hospitalier Annecy Genevois, Epagny Metz-Tessy, France; 44 Service de Médecine Intensive Réanimation, Centre Hospitalier Universitaire Gabriel-Montpied, Clermont-Ferrand, France; 45 Service de Médecine Intensive Réanimation, Hôpital Charles Nicolle, Centre Hospitalier Universitaire de Rouen; Normandie Université, UNIROUEN, Inserm U1096, FHU-REMOD-VHF, Rouen, France; 46 Service de Médecine Intensive Réanimation, Centre Hospitalier de Lens, Lens, France; 47 Service de Médecine Intensive Réanimation, Groupe Hospitalier Sud Ile de France, Melun, France; 48 Service de Réanimation Chirurgicale, Centre Hospitalier Universitaire Amiens-Picardie, Amiens, France; 49 Service de Médecine Intensive Réanimation, Centre Hospitalier du Mans, Le Mans, France; 50 Service de Médecine Intensive Réanimation, Centre Hospitalier Départemental de la Vendée, La Roche sur Yon, France; 51 Service de Médecine Intensive Réanimation, Centre Hospitalier de Béthune, Béthune, France; 52 Department of Anaesthesiology and Critical Care Medicine, Dijon University Medical Centre, Dijon, France; 53 Service de Médecine Intensive Réanimation, Centre Hospitalier Universitaire Hôtel-Dieu, 30 Bd. Jean Monnet, 44093 Nantes Cedex 1, France; 54 Laboratoire Psy-DREPI, Université de Bourgogne Pôle Aafe, Esplanade Erasme, 21078 Dijon, France

**Keywords:** Critical illness, Resilience, Post-traumatic stress disorder, Quality of life, Social support, Illness perception

## Abstract

**Background:**

Critical-illness survivors may experience post-traumatic stress disorder (PTSD) and quality-of-life impairments. Resilience may protect against psychological trauma but has not been adequately studied after critical illness. We assessed resilience and its associations with PTSD and quality of life, and also identified factors associated with greater resilience.

**Methods:**

This prospective, multicentre, study in patients recruited at 41 French ICUs was done in parallel with the NUTRIREA-3 trial in patients given mechanical ventilation and vasoactive amines for shock. Three months to one year after intensive-care-unit admission, survivors completed the Connor-Davidson Resilience Scale (CD-RISC-25), Impact of Event-Revised scale for PTSD symptoms (IES-R), SF-36 quality-of-life scale, Multidimensional Scale of Perceived Social Support (MSPSS), and Brief Illness Perception Questionnaire (B-IPQ).

**Results:**

Of the 382 included patients, 203 (53.1%) had normal or high resilience (CD-RISC-25 ≥ 68). Of these resilient patients, 26 (12.8%) had moderate to severe PTSD symptoms (IES-R ≥ 24) vs. 45 (25.4%) patients with low resilience (*p* = 0.002). Resilient patients had higher SF-36 scores. Factors independently associated with higher CD-RISC-25 scores were higher MSPSS score indicating stronger social support (OR, 1.027; 95%CI 1.008–1.047; *p* = 0.005) and lower B-IPQ scores indicating a more threatening perception of the illness (OR, 0.973; 95%CI 0.950–0.996; *p* = 0.02).

**Conclusions:**

Resilient patients had a lower prevalence of PTSD symptoms and higher quality of life scores, compared to patients with low resilience. Higher scores for social support and illness perception were independently associated with greater resilience. Thus, our findings suggest that interventions to strengthen social support and improve illness perception may help to improve resilience. Such interventions should be evaluated in trials with PTSD mitigation and quality-of-life improvement as the target outcomes.

**Supplementary Information:**

The online version contains supplementary material available at 10.1186/s13054-024-04989-x.

## Background

With advances in intensive care medicine and increasing numbers of patients admitted to intensive care units (ICUs), the number of critical-illness survivors is growing steadily. Studies have shown that these patients are at high risk for physical, cognitive, and psychological impairments that may persist for months or years. One of the main adverse psychological effects of critical illness is post-traumatic stress disorder (PTSD), which affects 4–62% of patients [1–4]. Clinical symptoms of PTSD include intrusive thoughts and memories of the traumatic event, avoidance of reminders of the event, and hyperarousal symptoms such as irritability, impaired concentration, and hypervigilance [5]. These symptoms can persist for over five years after ICU discharge and are associated with impaired quality of life [6].

Psychological resilience is the ability to adapt positively to traumatic and stressful events, thereby protecting against mental ill-health [7]. Resilient people are more likely to develop effective coping strategies for handling adverse situations [8]. Studies in patients with cancer have shown that greater resilience was associated with less anxiety and depression [9, 10]. Resilience can change throughout life and is influenced by both external factors, such as social support, and internal factors, such as perception of the illness and treatment [11–15].

Although the psychological burden of critical illness has been extensively investigated in ICU survivors and their relatives, few studies have focussed on patient resilience. Among patients having survived critical illness or trauma, the proportion with normal-to-high resilience varied widely, from 28 to 76%, and greater resilience was associated with less mental ill-health, pain, physical complaints, and self-care difficulties [16]. These data raise the possibility that promoting resilience in critical-illness survivors may improve psychological and quality-of-life outcomes [17]. However, only a few small studies have assessed the prevalence and determinants of resilience in this population, and they varied regarding the tools used to measure resilience [18].

The primary objective of the prospective, multicentre, observational RESIREA cohort study reported here was to assess resilience in a large cohort of survivors of severe critical illness, using the well-validated 25-item Connor-Davidson Resilience Scale (CD-RISC-25). The secondary objectives were to assess potential associations linking resilience to PTSD symptoms and quality of life and to identify factors associated with the level of resilience.

## Methods

### Study design and oversight

RESIREA was a planned study conducted in parallel with the randomised controlled multicentre open-label NUTRIREA-3 trial designed to evaluate whether low-calorie low-protein feeding decreased day-90 mortality and/or time to ICU discharge readiness, compared to standard calorie-protein supplies, in adults receiving invasive mechanical ventilation and vasopressor support for shock (ClinicalTrials.gov Identifier: NCT03573739) [19, 20]. The NUTRIREA-3 study was supported by the Nantes University Hospital and funded by a 2017 *Programme Hospitalier de Recherche Clinique National* grant from the French Ministry of Health (#PHRC-17-0213). NUTRIREA-3 was approved by the competent ethics committee (*Comité de Protection des Personnes Sud-Méditerranée 2*, #2018-A00424-51).

RESIREA was a multicentre, prospective, observational, cohort study. Of the 61 ICUs participating in the NUTRIREA-3 trial, 41 accepted to also participate in the RESIREA study, including 22 (53.7%) in university hospitals (Additional File 1: RESIREA sites and contributors). Because of organisational constraints, the RESIREA start date was not the same in all 41 ICUs. However, in all ICUs, inclusions in RESIREA stopped at inclusion of the last NUTRIREA-3 trial patient. The RESIREA patients were interviewed by psychologists, who administered five pre-specified questionnaires. The RESIREA study was supported by the Nantes University Hospital. The RESIREA study protocol was approved by the ethics committee of the French Intensive Care Society (CE SRLF 18–19).

### Participants

Inclusion in the study occurred in two steps. First, in each participating ICU, consecutive patients included in NUTRIREA-3 were considered for inclusion in RESIREA. Inclusion and exclusion criteria were similar to those for NUTRIREA-3: adults (18 years or older) were eligible if they were receiving invasive mechanical ventilation, with an expected duration of at least 48 h after inclusion and initiation either within 24 h after or within 24 h before ICU admission, concomitantly with vasoactive therapy for shock, and if nutritional support was expected to be started within 24 h after intubation (or within 24 h after ICU admission when intubation occurred before ICU admission). Non-inclusion criteria were specific nutritional needs, such as pre-existing long-term home enteral or parenteral nutrition for chronic bowel disease; dying patient, not-to-be-resuscitated order, or other treatment-limitation decision at ICU admission; pregnancy, recent delivery, or lactation; adult under guardianship; and correctional facility inmate. Specific informed consent for inclusion in the RESIREA study was obtained from the patients, or from their next of kin in patients unable to consent. In the second step, which occurred at ICU discharge, the following non-inclusion criteria were applied: death in the ICU, insufficient fluency in French, persistent severe illness or severe cognitive impairment precluding questionnaire completion, and consent withdrawal.

### Data collection

The baseline features of each patient were recorded at inclusion in the NUTRIREA-3 trial. Infectious and non-infectious complications during the ICU stay, dialysis in the ICU, duration of invasive mechanical ventilation, ICU and hospital lengths of stay, and mortality were recorded according to the NUTRIREA-3 trial protocol [19, 20].

Marital status, employment status, number of children, and history of psychiatric disorders were obtained during phone interviews by trained psychologists who had no knowledge of the medical data of the patients. Each interview involved the administration of five scales and lasted about 40 min. The phone numbers used were those in the ICU medical files for each patient. When a call was unanswered, at least two further attempts were made during different days or weeks. Two interviews were planned initially, three months and one year after inclusion in NUTRIREA-3 and RESIREA. However, organisational issues and difficulties experienced by some patients with participating in two long interviews prompted us to aim for at least one interview in each patient. When two interviews were performed, the data obtained during the most recent interview were used for the main analysis. Thus, in all patients, data from a single interview were analysed.

### Outcomes

Resilience was assessed with the CD-RISC-25 [21]. Each item is answered using a 0–4 Likert scale. The total score can range from 0 to 100, with higher scores indicating greater resilience. The items are grouped into five sub-scales: personal competence, high standards, and tenacity (eight items; sub-score range, 0–32), trust in one’s instincts, tolerance of negative affect, and strengthening effects of stress (seven items; sub-score range, 0–28), positive acceptance of change and secure relationships (five items; sub-score range, 0–20), control (three items; sub-score range, 0–12), and spiritual influences (two items; sub-score range, 0–8). We defined low, normal, and high resilience as CD-RISC-25 scores ≤ 67, 68–92, and ≥ 93, respectively [21, 22]. Patients designated as “resilient” hereafter are those with scores ≥ 68; patients with scores below this cut-off are designated “non-resilient”.

PTSD symptoms were assessed with the Impact of Event Scale-Revised (IES-R). The symptoms are grouped into three sub-scores: intrusion (sub-score range, 0–32), avoidance (sub-score range, 0–32), and hyperarousal (sub-score range, 0–24). The total score can range from 0 to 88, with higher scores indicating greater symptom severity. We defined severe, moderate, and no PTSD symptoms as IES-R scores ≥ 33, 24–32, and ≤ 23, respectively [23, 24].

Health-related quality of life was assessed with the Short Form-36 (SF-36) [25, 26]. The 36 items investigate eight dimensions: bodily pain, general health, mental health, physical functioning, role emotional, role physical, vitality, and social functioning. Eight sub-scores can be calculated, each of them contributing in different proportions to the calculation of two scores (PCS: physical component summary and MCS: mental component summary), each ranging from 0 to 100. Higher PCS and MCS scores indicate better health-related quality of life.

Social support was assessed with the Multidimensional Scale of Perceived Social Support (MSPSS), a 12-item questionnaire measuring the perceived adequacy of social support received from three sources: family, friends, and significant other persons. Each item is rated on a 7-point Likert scale (from 1, strongly disagree to 7, very strongly agree). The total score can range from 12 to 84 and each sub-score from 4 to 28. Higher values indicate greater social support [27].

Perception of illness by the patients was assessed with the Brief Illness Perception Questionnaire (B-IPQ). A 0–10 scale is used to rate eight of the nine B-IPQ items including five items for the cognitive illness representation sub-score (perceived consequences, perception of the timeline, amount of perceived personal control, amount of control of the treatment, and identity; range, 0–50), two items for the emotional illness representation sub-score (concern about the illness and emotional response to the illness; range, 0–20), and one item for the illness comprehensibility sub-score (understanding of the illness; range, 0–10). An additional item assesses causal perceptions by asking patients to list the three most likely causes for their illness. The total B-IPQ score is the sum of the scores on the first eight items and can thus range from 0 to 80, with higher scores reflecting a more threatening perception of the illness [28].

Perceived social support and perception of illness were assessed as dimensions possibly contributing to resilience after critical illness.

### Statistical analysis

No reliable data were available for anticipating the difference between groups. Given the observational design, we planned to perform adjusted analyses and, therefore, required sufficient observations for the large number of covariates (about 40 continuous and 10 qualitative covariates). We consequently planned to recruit 400 survivors of severe critical illness.

Baseline characteristics were described as number and percentage for qualitative variables and as mean ± SD and median [interquartile range] for quantitative variables.

For the five questionnaires, responses to at least 75% of items was required for inclusion in the analysis. Mean imputation was performed for missing data.

We determined the percentage of resilient patients (CD-RISC-25 score > 68), with the 95% confidence interval (95%CI). We also determined the percentage of patients with PTSD symptoms, overall and in each severity category (≥ 33 and 24–32), with the 95%CIs. The correlation between the IES-R and CD-RISC-25 scores was assessed by estimating the Spearman correlation coefficient, its 95%CI, and the associated *p* value. The SF-36, MSPSS, and B-IPQ scores were compared in resilient vs. non-resilient patients by applying the Wilcoxon test.

To identify factors associated with resilience, we first performed univariate analyses using the chi-square or Fisher test for qualitative variables and the Wilcoxon test for quantitative variables. Logistic regression was then used for the multivariate analysis. Variables with significant differences by univariate analysis (*p* < 0.20) were included in the multivariate analysis.

The analyses were performed using SAS version 9.4 (SAS Institute, Cary, NC) and R version 3.3.1 (htpps://www.r-project.org).

## Results

### Patients

**Fig. 1:**
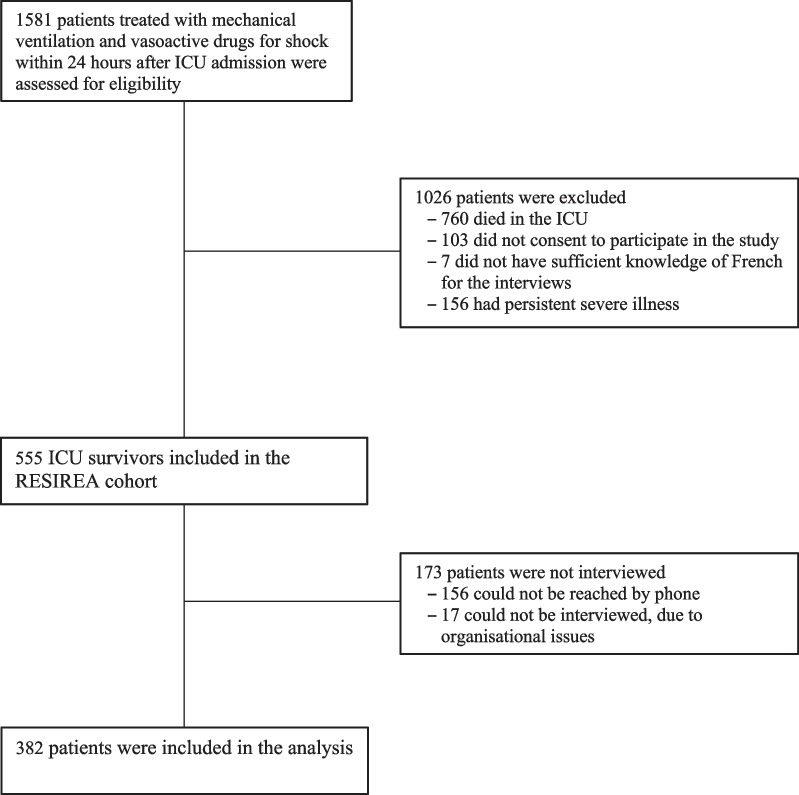
Flow chart. ICU: intensive care unit

Figure 1 is the patient flow chart. From 23 October 2018 to 8 December 2020, 1581 patients in the 41 participating ICUs were screened for eligibility; among them, 1026 had exclusion criteria, leaving 555 patients eligible for the RESIREA study. Of these, 173 were not interviewed. The remaining 382 (68.8%) patients were included in the analysis (Additional File 2, Table S1). Patients included in the analysis did not differ from patients who were eligible but could not be interviewed (Table 1).

**Table 1:** Comparison of the main baseline characteristics between patients included in the analysis and patients who were eligible for inclusion but were not interviewed

	Included patients N = 382	Eligible non-included patients N = 173	*p* value
Age, years, mean ± SD	61.4 ± 12.4	61.7 ± 14.5	0.55
Males, n (%)	257 (67.3)	115 (66.5)	0.85
McCabe score, n (%)			0.08
0 (no fatal underlying disease)	312 (81.7)	127 (73.4)	
1 (death expected within 5 years)	60 (15.7)	39 (22.5)	
2 (death expected within 1 year)	10 (2.6)	7 (4.0)	
Pre-existing illness at ICU admission, n (%)	222 (58.6)	103 (60.2)	0.71
Chronic renal failure	21 (5.5)	19 (11.1)	0.02
Liver disease	28 (7.4)	17 (10.0)	0.30
Cardiovascular disease	55 (14.6)	28 (16.6)	0.54
Chronic respiratory failure	25 (6.6)	19 (11.2)	0.07
Neurologic disease	34 (9.0)	19 (11.1)	0.43
Cancer or immune deficiency	85 (22.5)	25 (14.6)	0.03
Oesophageal, gastric, or duodenal ulcer	19 (5.0)	7 (4.1)	0.64
Diabetes mellitus	73 (19.3)	44 (25.7)	0.09
Weight, kg, median [IQR]	80.0 [68.0–92.0]	77.0 [67.0–89.5]	0.14
BMI, kg/m^2^, median [IQR]	27.3 [24.2–31.7]	26.6 [22.7–31.2]	0.15
SAPS II^a^, median [IQR]	55 [43–69]	59.0 [44.0–70.0]	0.32
SOFA score^b^, median [IQR]	10.0 [8.0–12.0]	10.0 [8.0–12.0]	0.06
Medical diagnosis at admission, n (%)	301 (78.8)	137 (79.2)	0.92
Acute illness at ICU admission, n (%)			0.09
Cardiac arrest	59 (15.5)	17 (9.8)	
Acute heart failure	65 (17.0)	28 (16.2)	
Acute central nervous system failure	25 (6.5)	13 (7.5)	
Acute respiratory failure	157 (41.1)	70 (40.5)	
Trauma	8 (2.1)	11 (6.4)	
Miscellaneous	68 (17.8)	34 (19.7)	
Cause of shock, n (%)			0.03
Cardiac	84 (22.0)	22 (12.7)	
Sepsis	206 (53.9)	102 (59.0)	
Other	92 (24.1)	49 (28.3)	
Ongoing treatments at inclusion, n (%)			
Randomised in the Low group of NUTRIREA-3^c^	176 (46.1)	95 (54.9)	0.05
Prone position	21 (5.5)	9 (5.2)	0.89
Sedative agents	348 (91.1)	156 (90.2)	0.73
NMBA	139 (36.4)	46 (26.6)	0.02
Insulin	147 (38.5)	73 (42.2)	0.41
Anti-microbial treatment^d^	326 (85.3)	152 (87.9)	0.43
Dialysis	30 (7.9)	19 (11.0)	0.23
Outcomes			
RRT during the ICU stay, n (%)	76 (19.9)	45 (26.0)	0.11
At least one complication^e^ during the ICU stay, n (%)	50 (13.1)	20 (11.6)	0.62
Duration of mechanical ventilation, days, median [IQR]	6.0 [2.0–11.0]	6.0 [3.0–10.0]	0.95
ICU length of stay, days, median [IQR]	9.0 [6.0–16.0]	9.0 [5.0–15.0]	0.91
Hospital length of stay, days, median [IQR]	21.0 [13.0–34.0]	20.0 [13.0–40.0]	0.29

*ICU* intensive care unit, *IQR* interquartile range, *BMI* body mass index, *SAPS II* Simplified Acute Physiology Score version II [29], *SOFA* Sequential Organ Failure Assessment [30], *NMBA* neuromuscular blocking agent, *RRT* renal replacement therapy

^a^SAPS II values can range from 0 (lowest level of critical illness) to 163 (most severe level of critical illness with 100% predicted mortality). A score of 50 predicts a 46.1% risk of death. The SAPS II was determined 24 h after ICU admission

^b^SOFA scores can range from 0 (no organ failure) to 24 (most severe level of multi-organ failure). The SOFA sub-score values at ICU admission are reported in eTable 1

^c^Patients included in the NUTRIREA-3 trial were randomised to early nutrition with either low or standard calorie-protein targets (6 kcal/kg/d and 0·2–0·4 g/kg/d, respectively; and 25 kcal/kg/d and 1·0–1·3 g/kg/d, respectively)

^d^Anti-microbial treatments included antibiotics, antiviral drugs, and antifungal drugs

^e^Complications included infections and gastro-intestinal complications acquired during the ICU stay

### Resilience, post-traumatic stress disorder symptoms, and quality of life

The median CD-RISC-25 was 69.0 [59.0–78.0]) (Table 2). Of the 382 patients, 203 (53.1%) were resilient, i.e., had CD-RISC-25 scores ≥ 68.

**Table 2:** Resilience in the 382 included patients discharged alive from the intensive care unit

	Median [IQR] or n (%)
CD-RISC-25^a^, median [IQR]	69.0 [59.0–78.0]
Personal competence, high standards, and tenacity	24.0 [20.0–27.0]
Trust in one’s instincts, tolerance of negative affect, and strengthening effects of stress	18.0 [15.0–21.0]
Positive acceptance of change, and secure relationships	15.0 [13.0–17.0]
Control	9.0 [7.0–10.0]
Spiritual influences	4.0 [3.0–6.0]
Resilience^b^, n (%)	
Low	179 (46.9)
Normal	195 (51.0)
High	8 (2.1)

*CD-RISC* Connor-Davidson Resilience Scale, *IQR* interquartile range

^a^The CD-RISC-25 has 25 items, with five sub-scales: personal competence, high standards, and tenacity (range, 0–32), trust in one’s instincts, tolerance of negative affect, and strengthening effects of stress (range, 0–28), positive acceptance of change and secure relationships (range, 0–20), control (range, 0–12), and spiritual influences (range, 0–8). Thus, the total CD-RISC-25 score can range from 0 to 100. Higher scores indicate greater resilience [21]

^b^Low, normal, and high resilience were defined as CD-RISC-25 scores ≤ 67, 68–92, and ≥ 93, respectively [21, 22]

The median total IES-R score was 9.0 [4.0–19.0]) (Table 3). The total score and each of the three sub-scores were significantly lower in resilient than in non-resilient patients. The IES-R score decreased as the CD-RISC-25 score increased (r, − 0.24; 95%CI [− 0.33 to − 0.14]; *p* < 0.0001; Additional File 3, Figure S1). Comparisons of CD-RISC-25 and IES-R scores obtained at 3 months vs. 12 months revealed no significant differences in the prevalence of resilient patients or in the prevalence of patients with PTSD (Additional File 4, Table S2).The median SF-36 PCS and MCS scores were 43.0 [34.0–51.0]) and 51.0 [40.0–57.0], respectively (Table 4). The MCS, PCS and seven of the eight sub-scores (the exception being role physical) were higher in resilient patients, indicating better quality of life compared to non-resilient patients.

**Table 3:** Post-traumatic stress disorder symptoms and quality of life in the 382 included patients discharged alive from the intensive care unit

	Overall population N = 382	Resilient patients^c^ N = 203	Non-resilient patients^c^ N = 179	*p* value
Post-traumatic stress disorder symptoms				
IES-R score^a^, median [IQR]	9.0 [4.0–19.0]	7.0 [3.0–15.0]	12.0 [5.0–24.0]	< 0.0001
Intrusion	4.0 [1.0–9.0]	3.0 [1.0–8.0]	5.0 [1.0–10.0]	0.016
Avoidance	3.0 [1.0–7.0]	2.0 [1.0–6.0]	5.0 [1.0–10.0]	0.0003
Hyperarousal	1.0 [0.0–4.0]	1.0 [0.0–3.0]	2.0 [0.0–6.0]	< 0.0001
PTSD symptoms, n (%)				
None	309 (81.3)	177 (87.2)	132 (74.6)	0.004
Moderate	29 (7.6)	13 (6.4)	16 (9.0)	
Severe	42 (11.1)	13 (6.4)	29 (16.4)	
Quality of life				
SF-36^b^, median [IQR]				
Bodily pain	72.0 [41.0–100.0]	84.0 [51.0–100.0]	62.0 [41.0–100.0]	0.006
General health	57.0 [45.0–72.0]	67.0 [52.0–80.0]	52.0 [40.0–62.0]	< 0.0001
Mental health	72.0 [56.0–84.0]	76.0 [64.0–88.0]	64.0 [48.0–80.0]	< 0.0001
Physical functioning	75.0 [45.0–95.0]	80.0 [55.0–95.0]	60.0 [35.0–90.0]	0.002
Role-emotional	100.0 [0.0–100.0]	100.0 [33.0–100.0]	67.0 [0.0–100.0]	0.0005
Role-physical	50.0 [0.0–100.0]	50.0 [0.0–100.0]	25.0 [0.0–100.0]	0.32
Vitality	55.0 [40.0–70.0]	60.0 [45.0–75.0]	45.0 [30.0–60.0]	< 0.0001
Social functioning	100.0 [63.0–100.0]	100.0 [63.0–100.0]	94.0 [50.0–100.0]	0.001
PCS	43.0 [34.0–51.0]	44.0 [34.0–52.0]	41.0 [33.0–49.0]	0.026
MCS	51.0 [40.0–57.0]	53.0 [45.0–59.0]	48.0 [35.0–54.0]	< 0.0001

*IES-R* Impact of Event Scale-Revised, *IQR* interquartile range, *PTSD* post-traumatic stress disorder, *SF-36* Short Form 36, *PCS* Physical Component Summary, *MCS* Mental Component Summary

^a^The IES-R score can range from 0 to 88 (intrusion sub-score, 0–32; avoidance sub-score, 0–32; hyperarousal sub-score, 0–24). A higher score indicates greater symptom severity [23]. IES-R scores ≥ 33, 24–32, and ≤ 23 indicate severe, moderate and no PTSD, respectively

^b^SF-36 is a 36-item health-related quality-of-life questionnaire that produces two scores (PCS and MCS) and the eight sub-scores listed in the table, each of which can range from 0 to 100. Higher scores indicate better health-related quality of life

^c^Resilient patients had CD-RISC-25 ≥ 68. Non-resilient patients had CD-RISC-25 ≤ 67

**Table 4:** Social support and illness perception in the 382 included patients discharged alive from the intensive care unit

	Overall population N = 382	Resilient patients^c^ N = 203	Non-resilient patients^c^ N = 179	*p* value
MSPSS^a^, median [IQR]	75.0 [63.0–83.0]	79.0 [69.0–84.0]	71.0 [59.0–79.0]	< 0.0001
Family	27.0 [23.0–28.0]	28.0 [25.0–28.0]	25.0 [20.0–28.0]	< 0.0001
Friends	28.0 [24.0–28.0]	25.0 [20.0–28.0]	22.0 [13.0–28.0]	< 0.0001
Significant other persons	24.0 [17.0–28.0]	28.0 [26.0–28.0]	27.0 [23.0–28.0]	< 0.0001
B-IPQ^b^, median [IQR]	37.0 [26.0–45.0]	32.0 [23.0–42.0]	40.0 [31.0–50.0]	< 0.0001
Cognitive illness representation	21.1 [15.0–28.0	20.0 [12.0–26.0]	25.0 [18.0–31.0]	< 0.0001
Emotional illness representation	12.0 [8.0–15.0]	10.0 [7.0–15.0]	13.5 [10.0–16.0]	< 0.0001
Illness comprehensibility	2.0 [0.0–5.0]	2.0 [0.0–3.0]	2.0 [1.0–5.0]	0.009

*ICU* intensive care unit, *MSPSS* Multidimensional Scale of Perceived Social Support, *B-IPQ* Brief Illness Perception Questionnaire, *IQR* interquartile range

^a^The total MSPSS score can range from 12 to 84 and each sub-score from 4 to 28. Higher values indicate stronger social support

^b^The total B-IPQ score can range from 0 to 80. The sub-scores for cognitive illness representation, emotional illness representation, and illness comprehensibility can range from 0 to 50, 0 to 20, and 0 to 10, respectively. A higher score reflects a more threatening perception of the illness

^c^Resilient patients had CD-RISC-25 ≥ 68. Non-resilient patients had CD-RISC-25 ≤ 67

### Factors associated with resilience

The median MSPSS and B-IPQ scores were 75.0 [63.0–83.0] and 37.0 [26.0–45.0], respectively (Table 4). Resilient patients had higher MSPSS score and sub-scores, indicating stronger perceived social support, and lower B-IPQ score and sub-scores, indicating more favourable perceptions of their illness, compared to non-resilient patients. By univariate analysis, resilient and non-resilient patients did not differ regarding baseline characteristics or ICU outcomes (Table 5). Both stronger perceived social support and a more favourable perception of illness were independently associated with resilience (MPSS: OR, 1.027; 95%CI 1.008–1.047; *p* = 0.005; B-IPQ: OR, 0.973; 95%CI 0.950–0.996; *p* = 0.02) (Additional File 5, Table S3).

**Table 5:** Univariate analysis of demographic characteristics in resilient and non-resilient patients

	Resilient patients^a^ N = 203	Non-resilient patients^a^ N = 179	*p* value
Age, years, mean ± SD	60.9 ± 12.9	62.0 ± 11.9	0.63
Males, n (%)	137 (67.5)	120 (67.0)	0.92
Marital status, n (%)			0.97
Single	34 (18.9)	30 (17.9)	
Married or living with a partner	113 (62.8)	107 (63.7)	
Divorced, separated, or widowed	33 (18.3)	31 (18.5)	
Employment status, n (%)			0.46
Employed	89 (49.4)	76 (45.5)	
Retired	91 (50.6)	91 (54.5)	
Children, n, median [IQR]	2 [0; 3]	2 [1; 3]	0.57
History of psychiatric disorder	13 (21.0)	4 (11.4)	0.23
McCabe score, n (%)			0.87
0 (no fatal underlying disease)	164 (80.8)	148 (82.7)	
1 (death expected within 5 years)	33 (16.3)	27 (15.1)	
2 (death expected within 1 year)	6 (3.0)	4 (2.2)	
Pre-existing illness at ICU admission, n (%)	114 (56.7)	108 (60.7)	0.43
Chronic renal failure	12 (6.0)	9 (5.1)	0.70
Liver disease	13 (6.5)	15 (8.4)	0.47
Cardiovascular disease	27 (13.5)	28 (15.7)	0.54
Chronic respiratory failure	12 (6.0)	13 (7.3)	0.59
Neurologic disease	18 (9.0)	16 (9.0)	0.99
Cancer or immune deficiency	43 (21.5)	42 (23.6)	0.63
Oesophageal, gastric, or duodenal ulcer	7 (3.5)	12 (6.7)	0.15
Diabetes mellitus	40 (19.9)	33 (18.5)	0.74
Weight, kg, median [IQR]	82.5 [71.0; 94.0]	76.0 [65.0; 90.7]	0.009
BMI, kg/m^2^, median [IQR]	28.1 [24.7; 33.1]	26.8 [23.3; 31.2]	0.02
SAPS II^b^, median [IQR]	53.0 [43.0; 70.0]	56.0 [44.0; 68.0]	0.43
SOFA score^c^, median [IQR]	10.0 [8.0; 12.0]	10.0 [8.0; 12.0]	0.85
Medical diagnosis at admission, n (%)	161 (79.3)	140 (78.2)	0.79
Acute illness at ICU admission, n (%)			0.57
Cardiac arrest	34 (16.7)	25 (14.0)	
Acute heart failure	36 (17.7)	29 (16.2)	
Acute central nervous system failure	9 (4.4)	16 (8.9)	
Acute respiratory failure	84 (41.4)	73 (40.8)	
Trauma	5 (2.5)	3 (1.7)	
Miscellaneous	35 (17.2)	33 (18.4)	
Cause of shock, n (%)			0.81
Cardiac	47 (23.2)	37 (20.7)	
Sepsis	109 (53.7)	97 (54.2)	
Other	47 (23.1)	45 (25.1)	
Ongoing treatments at inclusion, n (%)			
Randomised in the Low Group of NUTRIREA-3^d^	97 (47.8)	79 (44.1)	0.47
Prone position	14 (6.9)	7 (3.9)	0.20
Sedative agents	187 (92.1)	161 (89.9)	0.46
NMBA	78 (38.4)	61 (34.1)	0.38
Insulin	76 (37.4)	71 (39.7)	0.65
Anti-microbial treatment^e^	177 (87.2)	149 (83.2)	0.28
Dialysis	12 (5.9)	18 (10.1)	0.13
Outcomes			
RRT during the ICU stay, n (%)	36 (17.7)	40 (22.3)	0.26
At least one complication^f^ during the ICU stay, n (%)	24 (11.8)	26 (14.5)	0.43
Duration of mechanical ventilation, days, median [IQR]	5.0 [2.0; 10.0]	6.0 [3.0; 12.0]	0.32
ICU length of stay, days, median [IQR]	9.0 [5.0; 15.0]	10.0 [6.0; 16.0]	0.44
Hospital length of stay, days, median [IQR]	19.0 [12.0; 31.0]	22.0 [13.0; 35.0]	0.31
MSPSS score	79.0 [69.0; 84.0]	71.0 [59.0; 79.0]	< 0.0001
B-IPQ score	32.0 [23.0; 42.0]	40.0 [31.0; 50.0]	< 0.0001

*ICU* intensive care unit, *BMI* body mass index, *SAPS II* Simplified Acute Physiology Score version II, *SOFA* Sequential Organ Failure Assessment, *NMBA* neuromuscular blocking agent, *RRT* renal replacement therapy, *MSPSS* Multidimensional Scale of Perceived Social Support, *B-IPQ* Brief Illness Perception Questionnaire

^a^Resilient patients were defined as having a CD-RISC-25 score ≥ 68 and non-resilient patients as having a CD-RISC-25 score ≤ 67

^b^SAPS II values can range from 0 (lowest level of critical illness) to 163 (most severe level of critical illness with 100% predicted mortality). A score of 50 predicts a 46.1% risk of death. The SAPS II was determined 24 h after ICU admission

^c^SOFA scores can range from 0 (no organ failure) to 24 (most severe level of multi-organ failure). The SOFA sub-score values at ICU admission are reported in eTable 1

^d^Patients included in the NUTRIREA-3 trial were randomised to early nutrition with either low or standard calorie-protein targets (6 kcal/kg/d and 0·2–0·4 g/kg/d, respectively; and 25 kcal/kg/d and 1·0–1·3 g/kg/d, respectively)

^e^Anti-microbial treatments included antibiotics, antiviral drugs, and antifungal drugs

^f^Complications included infections and gastro-intestinal complications acquired during the ICU stay

## Discussion

In this large prospective study, half the survivors of severe critical illness had normal to high resilience. These resilient patients were less affected by PTSD symptoms and had higher health-related quality of life scores than did non-resilient patients. Resilience was associated neither with the characteristics of the acute illness nor with sex, age, or any other baseline variables. In contrast, stronger perceived social support and a more favourable perception of the illness were independently associated with resilience. Although causal interferences cannot strongly be assessed with our study design, these findings suggest interventions for increasing resilience with the goal of decreasing trauma-induced adverse responses and for improving quality of life in survivors of critical illness.

Critical illness is a traumatic event that can be followed by PTSD symptoms and quality-of-life impairments. Previous studies in non-critically ill patients have shown that interventions to improve resilience shortly after trauma exposure or in patients with PTSD may be useful to limit PTSD symptoms and improve quality of life [12]. A pilot randomised clinical trial showed that a brief six-session resilience-building programme started just before ICU discharge in survivors of critical neurological injury was feasible and associated with reduced symptoms of anxiety, depression, and PTSD three months later compared to minimally enhanced standard care in the patients and their informal caregivers [17]. This finding is important, as it supports the ability of resilience-building to prevent subsequent mental distress. The median IES-R score and proportion of patients with PTSD symptoms in our cohort are in agreement with earlier reports [1]. Importantly, PTSD symptoms were twice as common in the non-resilient group as in the resilient group, in keeping with a study in trauma patients [16]. Resilient patients also had higher quality of life scores as compared to non-resilient patients. Moreover, given that 47% of patients had low resilience, these findings suggest that improving resilience may be associated with a reduction in PTSD and improved quality of life in survivors of critical illness.

Resilience was not associated neither with the characteristics of the acute illness nor with the baseline features of the patients. This finding is consistent with a previous study in trauma patients [16]. In contrast the independent associations with social support and illness perception suggest avenues for intervention. The effect size seems small in our study, but others have also reported associations linking mental health, social support and resilience [16, 31]. Moreover perception of illness was associated with psychological well-being and improved coping [32–34]. A highly positive perception of illness was associated with greater treatment adherence and improved recovery and mental health [35, 36]. Illness perception is probably amenable to modification via psychological support [37]. Programmes have been developed in settings other than critical illness to increase the perception of control over the disease [38]. In a randomised controlled trial in patients with heart failure, an educational programme was effective in improving illness perception and was also associated with better results on measures of quality of life and self-care [39]. Improving illness perception resulted in better outcomes of patients with chronic low back pain in another randomised controlled trial [40]. Illness perception seems readily amenable to change via simple interventions, which thus deserve to be investigated in survivors of critical illness [41]. In the ICU, research is needed to determine whether assessing illness perception may help intensivists to apply communication techniques likely to improve their patients’ understanding of their illness and experience in the ICU. Finally, our study suggests that interventions focused on promoting resilience may be more effective than targeting specific disorders in ICU survivors. Qualitative studies showed that patients experienced difficulties in accessing the appropriate care for post-ICU syndrome, obtaining information about their post-resuscitation symptoms, and understanding what they had experienced in the ICU [42, 43]. Our study showing high levels of PTSD symptoms supports the development of post-ICU follow-up visits and the routine provision of psychologist support after ICU discharge. Assessment of social support and illness perception may allow multidisciplinary interventions aimed at improving resilience, thereby alleviating PTSD symptoms and improving quality of life [44].

Our study has several limitations. First, that 31% of patients eligible for the study could not be interviewed may have introduced selection bias. However, we evidenced no significant differences between these patients and those who were interviewed. Moreover, to our knowledge, RESIREA is the largest study to date of resilience after critical illness. Second, logistical issues prevented us from performing two interviews, three months and one year after ICU discharge, in all patients. Also, we had no data on resilience or PTSD symptoms before the critical illness. We were consequently unable to assess possible changes in these two variables over time in individual patients. However, data collected at 3 and at 12 months did not differ significantly regarding the proportions of resilient patients or of patients with PTSD symptoms. These findings align with a scoping review showing no clinically significant changes in mental-health symptoms over the first year after ICU discharge, also with no pre-illness data [18]. Using both 3-month and 12-month data in our study was therefore legitimate and cannot have affected the results, their interpretation, or our conclusion. Importantly, the 53.1% proportion of resilient patients was within the previously reported range (28–67%) in critical-care settings [18]. Third, all 41 participating ICUs were in France. This may limit the general applicability of our findings. Nonetheless, the strengths of our study include the prospective design, multicentre recruitment providing a large sample size, and use of the CD-RISC-25 to assess resilience. This tool has well-validated psychometric properties [18, 21]. Also, the data were collected by psychologists trained in telephone interviewing and in administration of the five questionnaires used for the study. Questionnaires data were missing for less than 2% of patients. These facts support the reliability and general applicability of our data. Fourth, the observational design of the study precludes definitive conclusions about causal relationships between resilience and PTSD in our cohort. Measurements at baseline then repeatedly during follow-up would be ideal to assess causality. Nonetheless, our findings generate strong hypotheses for designing interventional trials aimed at enhancing resilience and thereby possibly mitigating PTSD and improving quality of life. Last, we focussed on patients who survived an episode of severe critical illness requiring at least invasive mechanical ventilation and vasoactive drugs. We thus studied a uniform population of patients at high risk for long-term mental-health disturbances and, therefore, likely to benefit the most from strategies designed to improve resilience.

## Conclusions

Among survivors of severe critical illness, those with normal or high resilience were less affected by PTSD symptoms and had higher health-related quality-of-life scores. Greater resilience was independently associated with stronger social support and a more favourable perception of the illness but not with the characteristics of the acute illness or the baseline variables. Attention to social support and illness perception may help to strengthen resilience with the goal of improving the psychological outcomes of ICU survivors. `

### Supplementary Information

Supplementary Material 1.

Supplementary Material 2.

Supplementary Material 3.

Supplementary Material 4.

Supplementary Material 5.

## Data Availability

The datasets used and/or analysed during the current study are available from the corresponding author on reasonable request.

## Abbreviations

PTSD: Post-traumatic stress disorder
ICU: Intensive Care Unit
CD-RISC-25: Connor-Davidson Resilience Scale
IES-R: Impact of Event-Revised scale
SF-36: Short Form-36
PCS: Physical component summary
MCS: Mental component summary
MSPSS: Multidimensional Scale of Perceived Social Support
B-IPQ: Brief Illness Perception Questionnaire
