# Supplementary material for: Resilience after severe critical illness: a prospective, multicentre, observational study (RESIREA)
Source: Crit Care. 2024 Jul 12;28:237. doi: 10.1186/s13054-024-04989-x (PMC11245798; doi:10.1186/s13054-024-04989-x)
Supplement: Supplementary file 1 — Supplementary Material 1. [file 13054_2024_4989_MOESM1_ESM.docx]

**Additional File 1:** **RESIREA sites and contributors**

1. Laboratoire Psy-DREPI, Université de Bourgogne Pôle Aafe, Esplanade Erasme, 21078 Dijon, France: Alice Mathieu, Alexandra Laurent
2. Inserm CIC 1415, Tours, France: Amélie Le Gouge
3. Service de Médecine Intensive Réanimation, Centre Hospitalier Universitaire de Nantes, Nantes, France; Université de Nantes, Nantes, France: Jean Reignier, MD, PhD; Jean-Baptiste Lascarrou, MD, PhD; Diane Maugars
4. Service de Médecine Intensive Réanimation, Centre Hospitalier d’Argenteuil, Argenteuil, France: Gaëtan Plantefève, MD, Damien Contou, MD, Olivier PAJOT, MD, Cécile Le Parco, RN
5. Service de Médecine Intensive Réanimation, Hôpital Cochin, Groupe Hospitalier Paris Centre-Université Paris Cité, Assistance Publique –Hôpitaux de Paris, Paris, France: Jean-Paul Mira MD, PhD; Paul Jaubert, MD; Nathalie Marin, PharmD
6. Service de Médecine Intensive Réanimation, Hôpital Edouard Herriot, Hospices Civils de Lyon, Lyon, France: Laurent Argaud, MD, PhD; Marie Simon, MD; Martin Cour, MD, PhD; Marion Provent, RN
7. Service de Médecine Intensive Réanimation, Centre Hospitalier Universitaire Angers, Angers, France: Pierre Asfar, MD, PhD; Satar Mortaza, MD; Marc Pierrot, MD; Vincent Souday, MD; Marie Lemerle MD
8. Service de Médecine Intensive Réanimation, Hôpital Nord Franche Comté, Trevenans, France: Julio Badie, MD; Sylvain Malfroy, MD; Fernando Berdaguer Ferrari, MD
9. Service de Médecine Intensive Réanimation, Centre Hospitalier de Saint Malo, Saint-Malo, France: Nicolae-Vlad Botoc, MD
10. Service de Médecine Intensive Réanimation, Centre Hospitalier Universitaire de Bordeaux, Bordeaux, France: Hoang-Nam Bui, MD; Didier Gruson MD, PhD; Charline Sazio, MD; Suzanne Champion, MD
11. Service de Médecine Intensive Réanimation, Centre Hospitalier Universitaire de Poitiers, Poitiers, France: Rémi Coudroy, MD, PhD; Florence Boissier, MD, PhD; Anne Veinstein, MD
12. Service de Médecine Intensive Réanimation, Hôpital de la Croix Rousse, Hospices Civils de Lyon, Lyon, France: Louis Chauvelot, MD; Loredana Baboi, PhD; Jean-Christophe Richard, MD, PhD; Hodane Yonis, MD
13. Service de Médecine Intensive Réanimation, Centre Hospitalier d’Angoulême, Angoulême, France: Christophe Cracco, MD; David Schnell, MD
14. Service de Médecine Intensive Réanimation, Centre Hospitalier Universitaire Saint Louis, Assistance Publique –Hôpitaux de Paris, Paris, France: Michael Darmon, MD; Virginie Lemiale, MD; Eric Mariotte, MD; Sandrine Valade, MD
15. Service de réanimation polyvalente, Centre Hospitalier Bretagne-Atlantique, Vannes, France: Agathe Delbove, MD; Yannick Fedun, MD; Sébastien Bigot, RN
16. Service de Réanimation polyvalente, Hôpital Foch, Suresnes, France: Jerôme Devaquet, MD; Charles Cerf, MD; Guillaume Tachon, MD
17. Service de Médecine Intensive Réanimation, Hôpital Louis-Mourier, Assistance Publique –Hôpitaux de Paris, Colombes, France: Louis-Marie Dumont, MD
18. Service de Médecine Intensive Réanimation, Centre Hospitalier de Chartres, Chartres, France: Olivier Gontier, MD
19. Service de Médecine Intensive Réanimation, Centre Hospitalier de Montauban, Montauban, France: Samuel Groyer, MD; Jérôme Roustan, MD; Sylvie Vimeux, MD; Michel Bonnivard, MD
20. CHU Nantes, INSERM, Nantes Université, Anesthesie Reanimation, CIC 1413, Nantes, France: Yannick Hourmant, MD; Karim Asehnoune MD, PhD; Antoine Roquilly MD, PhD
21. Service de Réanimation chirurgicale, Hôpital Saint-Eloi, CHU de Montpellier, Montpellier, France; PhyMedExp, INSERM, CNRS, Montpellier, France: Samir Jaber, MD, PhD; Fouad Belafia, MD; Matthieu Conseil, MD; Moussa Cisse, MD
22. Service de Médecine Intensive Réanimation, Centre Hospitalier de Valenciennes, Valenciennes, France: Lambiotte Fabien, MD; Bouras Chaouki, MD; Fodil Hannane, RN
23. Service de Médecine Intensive Réanimation, Centre Hospitalier de Bigorre, Tarbes, France: Jeremy Castanera, MD; Thiery Dulac, MD; Philippe Petua, MD
24. Service de Médecine Intensive Réanimation, Centre Hospitalier Universitaire Amiens-Picardie, Amiens, France: Julien Maizel MD, PhD; Yoann Zerbib MD; Clement Brault MD
25. Service de Médecine Intensive Réanimation, Centre Hospitalier Universitaire de la Réunion, Saint-Denis, La Réunion, France: Olivier Martinet, MD
26. Service de Médecine Intensive Réanimation, Hôpital Raymond Poincaré, Assistance Publique-Hôpitaux de Paris, Garches, France; Inserm U 1173, Université de Versailles-Saint Quentin en Yvelines, Versailles, France: Djillali Annane ,MD PhD; Rania Bounab, MD; Nicholas Heming, MD PhD;Virginie Maxime, MD
27. Service de Médecine Intensive Réanimation, Centre Hospitalier Universitaire de Tours, CRICS-TRIGGERSEP network Tours, France: Emmanuelle Mercier, MD
28. Service de Médecine Intensive Réanimation, Centre Hospitalier Universitaire d’Orléans, Orléans, France: Mai-Anh Nay, MD; Thierry Boulain, MD; Sophie Jacquier, MD; Grégoire Muller, M​D
29. Médecine Intensive-Réanimation, CHU Lille, F-59000; CNRS, Inserm, UMR 8576 - U1285 - UGSF - Unité de Glycobiologie Structurale et Fonctionnelle, Univ. Lille, France: Saad Nseir, MD, PhD, Raphaël Favory, MD, PhD, Sébastien Preau, MD, PhD, Julien Poissy, MD, PhD
30. Service de Médecine Intensive Réanimation, Centre Hospitalier Universitaire de Besançon, Besançon, France: Gaël Piton, MD, PhD; Hadrien Winiszewski, MD; Thibault Vieille, MD
31. Service de Médecine Intensive Réanimation, Centre Hospitalier Universitaire François Mitterrand, Dijon, France; Lipness Team, INSERM, LabExLipSTIC, Université de Bourgogne, Dijon, France; INSERM Centres d'Investigation Clinique, Département d’épidémiologie clinique, Université de Bourgogne, Dijon, France: Jean-Pierre Quenot, MD, PhD; Marine Jacquier, MD; Marie Labruyère, MD; Pascal Andreu, MD
32. Service de Médecine Intensive Réanimation, Centre Hospitalier Universitaire La Cavale Blanche, Brest, France: Anne Renault, MD; Laetitia Bodenes, MD
33. Service de Médecine Intensive Réanimation, Centre Hospitalier de Dieppe, Dieppe, France: Jean-Philippe Rigaud, MD, PhD; Antoine Marchalot, MD; Cécile Lafosse, RN; Christelle Bigot, RN
34. Service de Médecine Intensive Réanimation, Hôpitaux Universitaires de Strasbourg, Strasbourg, France: Françis Schneider, MD, PhD; Jean-Etienne Herbrecht, MD; Raphaël Clere-Jehl, MD, PhD
35. Service de Médecine Intensive Réanimation, Centre Hospitalier Annecy Genevois, Epagny Metz-Tessy, France: Michel Sirodot, MD; David Bougon, MD; Etienne Escudier, MD; Marion Lebouc, SC
36. Service de Médecine Intensive Réanimation, Centre Hospitalier Universitaire Gabriel-Montpied, Clermont-Ferrand, France: Bertrand Souweine, MD, PhD; Elisabeth Coupez, MD; Claire Dupuis, MD, PhD
37. Service de Médecine Intensive Réanimation, Hôpital Charles Nicolle, Centre Hospitalier Universitaire de Rouen; Normandie Université, UNIROUEN, Inserm U1096, FHU-REMOD-VHF, Rouen, France: Fabienne Tamion, MD,PhD; Gregoire Jolly MS, Zoé Demailly MD
38. Service de Médecine Intensive Réanimation, Centre Hospitalier de Lens, Lens, France: Didier Thévenin, MD
39. Service de Médecine Intensive Réanimation, Groupe Hospitalier Sud Ile de France, Melun, France: Nathalie Thieulot-Rolin, MD; Jonathan Chelly, MD; Franck Pourcine, MD; Ly Van Vong, MD
40. Service de Réanimation Chirurgicale, Centre Hospitalier Universitaire Amiens-Picardie, Amiens, France: François Tinturier, MD
41. Service de Médecine Intensive Réanimation, Centre Hospitalier du Mans, Le Mans, France: Patrice Tirot, MD; Christophe Guitton, MD; Nicolas Chudeau, MD; Mickaël Landais , MD
42. Service de Médecine Intensive Réanimation, Centre Hospitalier Départemental de la Vendée, La Roche sur Yon, France: Isabelle Vinatier, MD; Angélique Deschamps, RN; Natacha Maquigneau, RN
43. Service de Médecine Intensive Réanimation, Centre Hospitalier de Béthune, Béthune, France: Christophe Vinsonneau, MD; Caroline Sejourne, MD; Imen Rahmani, MD; Ghada Sboui, MD
