# Supplementary material for: Resilience after severe critical illness: a prospective, multicentre, observational study (RESIREA)
Source: Crit Care. 2024 Jul 12;28:237. doi: 10.1186/s13054-024-04989-x (PMC11245798; doi:10.1186/s13054-024-04989-x)
Supplement: Supplementary file 2 — Supplementary Material 2. [file 13054_2024_4989_MOESM2_ESM.docx]

**Additional File 2**

**Table S1: Main features of the 382 included patients discharged alive from the intensive care unit**

|  | **mean±SD, median [IQR], or n (%)** |
| --- | --- |
| Age, years, mean±SD | 61.4 ± 12.4 |
| Males, n (%) | 257 (67.3) |
| Marital status, n (%) |  |
| Single | 64 (18.4) |
| Married or living with a partner | 220 (63.2) |
| Divorced, separated, or widowed | 64 (18.4) |
| Employment status, n (%) |  |
| In work | 165 (47.6) |
| Retired | 182 (52.4) |
| Number of children, median [IQR] | 2 [1–3] |
| History of psychiatric disorder | 17 (4.5) |
| McCabe score, n (%) |  |
| 0 (no fatal underlying disease) | 312 (81.7) |
| 1 (death expected within 5 years) | 60 (15.7) |
| 2 (death expected within 1 year) | 10 (2.6) |
| Pre-existing illness at ICU admission, n (%) | 222 (58.6) |
| Chronic renal failure | 21 (5.5) |
| Liver disease | 28 (7.4) |
| Cardiovascular disease | 55 (14.6) |
| Chronic respiratory failure | 25 (6.6) |
| Neurologic disease | 34 (9.0) |
| Cancer or immune deficiency | 85 (22.5) |
| Oesophageal, gastric, or duodenal ulcer | 19 (5.0) |
| Diabetes mellitus | 73 (19.3) |
| Weight, kg, median [IQR] | 80.0 [68.0–92.0] |
| BMI, kg/m², median [IQR] | 27.3 [24.2–31.7] |
| SAPS II^a^, median [IQR] | 55 [43–69] |
| SOFA score^b^, median [IQR] | 10.0 [8.0–12.0] |
| Medical diagnosis at admission, n (%) | 301 (78.8) |
| Acute illness at ICU admission, n (%) |  |
| Cardiac arrest | 59 (15.5) |
| Acute heart failure | 65 (17.0) |
| Acute central nervous system failure | 25 (6.5) |
| Acute respiratory failure | 157 (41.1) |
| Trauma | 8 (2.1) |
| Miscellaneous | 68 (17.8) |
| Cause of shock, n (%) |  |
| Cardiac | 84 (22.0) |
| Sepsis | 206 (53.9) |
| Other | 92 (24.1) |
| Ongoing treatments at inclusion, n (%) |  |
| Randomised to the Low Group of NUTRIREA-3^c^ | 176 (46.1) |
| Prone position | 21 (5.5) |
| Sedative agents | 348 (91.1) |
| NMBA | 139 (36.4) |
| Insulin | 147 (38.5) |
| Anti-microbial treatment^d^ | 326 (85.3) |
| Dialysis | 30 (7.9) |
| Outcomes |  |
| RRT during the ICU stay, n (%) | 76 (19.9) |
| One or more complications^e^ in the ICU, n (%) | 50 (13.1) |
| Days on mechanical ventilation, median [IQR] | 6.0 [2.0 ; 11.0] |
| ICU length of stay, days, median [IQR] | 9.0 [6.0 ; 16.0] |
| Hospital length of stay, days, median [IQR] | 21.0 [13.0 ; 34.0] |

ICU: intensive care unit; IQR: interquartile range; BMI: body mass index; SAPS II: Simplified Acute Physiology Score version II; SOFA: Sequential Organ Failure Assessment; NMBA: neuromuscular blocking agent; RRT: renal replacement therapy

^a^SAPS II values can range from 0 (lowest level of critical illness) to 163 (most severe level of critical illness with 100% predicted mortality). A score of 50 predicts a 46.1% risk of death. The SAPS II was determined 24 hours after ICU admission.

^b^SOFA scores can range from 0 (no organ failure) to 24 (most severe level of multi-organ failure). The SOFA sub-score values at ICU admission are reported in eTable 1.

^c^ Patients included in the NUTRIREA-3 trial were randomised to early nutrition with either low or standard calorie-protein targets (6 kcal/kg/d and 0·2–0·4 g/kg/d, respectively; and 25 kcal/kg/d and 1·0–1·3 g/kg/d, respectively).

^d^ Anti-microbial treatments included antibiotics, antiviral drugs, and antifungal drugs.

^e^ Complications included infections and gastro-intestinal complications acquired during the ICU stay.
