# Supplementary material for: Resilience after severe critical illness: a prospective, multicentre, observational study (RESIREA)
Source: Crit Care. 2024 Jul 12;28:237. doi: 10.1186/s13054-024-04989-x (PMC11245798; doi:10.1186/s13054-024-04989-x)
Supplement: Supplementary file 3 — Supplementary Material 3. [file 13054_2024_4989_MOESM3_ESM.docx]

**Additional File 3**

**Figure S1: Correlation between the CD-RISC-25 and IES-R scores**

R: -0.24, 95% confidence interval, -0.33 to -0.14; *p*<0.0001

Correlations between the CD-RISC and IES-R scores were similar in the groups interviewed 3 months vs. 12 months after enrolment in the NUTRIREA-3 and RESIREA studies: the correlation coefficients (R) for the 3-month and 12-month data were -0.29 (95% confidence interval, -0.38 to -0.19) and -0.23 (95% confidence interval, -0.35 to -0.10), respectively.

CD-RISC-25, 25-item Connor-Davidson Resilience Scale; IES-R: Impact of Event Scale-Revised
