# Supplementary material for: Resilience after severe critical illness: a prospective, multicentre, observational study (RESIREA)
Source: Crit Care. 2024 Jul 12;28:237. doi: 10.1186/s13054-024-04989-x (PMC11245798; doi:10.1186/s13054-024-04989-x)
Supplement: Supplementary file 4 — Supplementary Material 4. [file 13054_2024_4989_MOESM4_ESM.docx]

**Additional File 4**

**Table S2: Resilience, post-traumatic stress disorder, quality of life, social support and illness perception 3 and 12 months after inclusion in NUTRIREA-3 and RESIREA**

|  | **3 months**  **n1** | **12 months**  **n2** |
| --- | --- | --- |
| **Resilience^b^, n (%)** (n1=333, n2=222) |  |  |
| Low | 153 (45.9) | 105 (47.3) |
| Normal | 171 (51.4) | 113 (50.9) |
| High | 9 (2.7) | 4 (1.8) |
| **Post-traumatic stress disorder symptoms*,* n (%)** (n1=333, n2=224) |  |  |
| None | 261 (78.4) | 185 (82.6) |
| Moderate | 28 (8.4) | 17 (7.6) |
| Severe | 44 (13.2) | 22 (9.8) |
| **Quality of life** (n1=314, n2=209) |  |  |
| PCS | 39.0 [32.0 ; 48.0] | 46.0 [35.0 ; 53.0] |
| MCS | 51.0 [39.0 ; 58.0] | 52.0 [43.0 ; 57.0] |
| **Social support** (n1=334, n2=222) |  |  |
| MSPSS score | 76.0 [64.0 ; 84.0] | 76.0 [64.0 ; 82.0] |
| **Illness perception** (n1=333, n2=222) |  |  |
| B-IPQ score | 37.0 [28.0 ; 46.0] | 36.0 [25.0 ; 44.0] |

ICU: intensive care unit; BMI: body mass index; SAPS II: Simplified Acute Physiology Score version II; SOFA: Sequential Organ Failure Assessment; NMBA: neuromuscular blocking agent; RRT: renal replacement therapy; MSPSS: Multidimensional Scale of Perceived Social Support; B-IPQ: Brief Illness Perception Questionnaire

^a^Resilient patients were defined as having a CD-RISC-25 score ≥68 and non-resilient patients as having a CD-RISC-25 score ≤67.

^b^SAPS II values can range from 0 (lowest level of critical illness) to 163 (most severe level of critical illness with 100% predicted mortality). A score of 50 predicts a 46.1% risk of death. The SAPS II was determined 24 hours after ICU admission.

^c^SOFA scores can range from 0 (no organ failure) to 24 (most severe level of multi-organ failure). The SOFA sub-score values at ICU admission are reported in eTable 1.

^d^Patients included in the NUTRIREA-3 trial were randomised to early nutrition with either low or standard calorie-protein targets (6 kcal/kg/d and 0·2–0·4 g/kg/d, respectively; and 25 kcal/kg/d and 1·0–1·3 g/kg/d, respectively).

^e^Anti-microbial treatments included antibiotics, antiviral drugs, and antifungal drugs.

^f^Complications included infections and gastro-intestinal complications acquired during the ICU stay.
