# Supplementary material for: Resilience after severe critical illness: a prospective, multicentre, observational study (RESIREA)
Source: Crit Care. 2024 Jul 12;28:237. doi: 10.1186/s13054-024-04989-x (PMC11245798; doi:10.1186/s13054-024-04989-x)
Supplement: Supplementary file 5 — Supplementary Material 5. [file 13054_2024_4989_MOESM5_ESM.docx]

**Additional File 5**

**Table S3: Factors independently associated with resilience by multivariate analysis; data were available for 361 patients**

|  | **OR** | **95%CI** | ***p* value** |
| --- | --- | --- | --- |
| MSPSS^a^ | 1.027 | [1.008–1.047] | 0.005 |
| B-IPQ score^b^ | 0.973 | [0.950–0.996] | 0.02 |

MSPSS, Multidimensional Scale of Perceived Social Support; B-IPQ, Brief Illness Perception Questionnaire

^a^The total MSPSS score can range from 12 to 84 and each sub-score from 4 to 28. Higher values indicate stronger social support.

^b^The B-IPQ score can range from 0 to 80. The cognitive illness representation, emotional illness representation, and illness comprehensibility sub-scores can range from 0 to 50, 0 to 20, and 0 to 10, respectively. Higher scores reflect a more threatening perception of the illness.
